# Prevalence of multimorbidity and its correlates among older adults in Eastern Nepal

**DOI:** 10.1186/s12877-022-03115-2

**Published:** 2022-05-16

**Authors:** Siva Balakrishnan, Isha Karmacharya, Saruna Ghimire, Sabuj Kanti Mistry, Devendra Raj Singh, Om Prakash Yadav, Nachiket Gudi, Lal Bahadur Rawal, Uday Narayan Yadav

**Affiliations:** 1 School of Public Health, University of Nevada Reno, Reno, NV USA; 2 Department of Sociology and Gerontology and Scripps Gerontology Center, Miami University, Oxford, OH USA; 3 Centre for Primary Health Care and Equity, University of New South Wales, Sydney, Australia; 4 School of Human and Health Sciences, University of Huddersfield, Queensgate, Huddersfield, UK; 5 Ministry of Health and Population, Kathmandu, Nepal; 6 Public Health Evidence South Asia (PHESA), Department of Health Information, Prasanna School of Public Health, Manipal, Karnataka India; 7 School of Health Medical and Applied Sciences, College of Science and Sustainability, Central Queensland University, Sydney Campus, Sydney, Australia; 8 National Centre for Epidemiology and Population Health, The Australian National University, Canberra, Australia; 9 Centre for Research Policy and Implementation (CRPIN), Biratnagar, Nepal

**Keywords:** Nepal, Older adults, Multimorbidity, NCDs, Chronic diseases

## Abstract

**Background:**

The number of people with multimorbidity is surging around the world. Although multimorbidity has been introduced in policy and practice in developed countries, developing countries like Nepal have not considered it as a matter of public health urgency due to the lack of enough epidemiological data. Multimorbidity profoundly affects older adults’ wellbeing; therefore, it is crucial to estimate its prevalence and determinants. This study aimed to estimate the prevalence of multimorbidity among older adults in Eastern Nepal and identify its correlates.

**Methodology:**

A community-based cross-sectional survey was conducted in three districts of Eastern Nepal. Data were collected between July and September 2020, among 847 Nepali older adults, aged 60 and older, where study participants were recruited through a multi-stage cluster sampling technique. Semi-structured interviews were conducted at the community settings to collect data. Logistic regression assessed correlates of multimorbidity. SAS 9.4 was used to run all statistical tests and analyses.

**Results:**

More than half (66.5%) of the participants had at least one of the five non-communicable chronic conditions; hypertension (31.6%), osteoarthritis (28.6%), chronic respiratory disease (18.0%), diabetes (13.5%), and heart disease (5.3%). The prevalence of multimorbidity was 22.8%. In the adjusted model, increased age (for 70–79 years, OR: 3.11, 95% CI: 1.87–5.18; for 80 + years, OR: 4.19, 95% CI: 2.32–7.57), those without a partner (OR: 1.52, 95% CI: 1.00–2.30), residing in urban areas (OR: 1.71, 95% CI: 1.16–2.51), and distant from health center (OR: 1.66, 95% CI: 1.04–2.64) were significantly associated with multimorbidity.

**Conclusions:**

This study found one in five study participants had multimorbidity. The findings will assist policymakers and stakeholders in understanding the burden of multimorbidity among the older population and identifying the groups in most need of health promotion intervention. Future interventions may include developing horizontal multimorbid approaches and multisectoral strategies specifically tailored to meet the needs of those populations.

## Background

Nepal, a South Asian country between India and China, is concurrently facing demographic and epidemiological transition. Demographically, the population of older adults, which comprises 8.1% (2.5 million) of the total population, is increasing at a rate (3.5%) greater than the national population growth rate (1.35%) [1]. The percentage of older adults aged 60 years and over is predicted to rise from 8.6 in 2015 to 10.8 in 2030 [2]. Epidemiologically, Nepal is transitioning from a historically high burden of infectious diseases to chronic non-communicable diseases. NCDs contributed to 58.7% of the total burden of disease in 2017 with cardiovascular disease, respiratory disease and cancer being the primary cause of death in the country [3]. The established risk factors for NCDs are highly prevalent, further contributing to future spikes in NCDs. The concurrent demographic and epidemiological transition mean that many people will survive into old age with one or more NCDs.

Notably, people living with NCDs often have multiple rather than a single condition [4]. Consequently, the number of people with multimorbidity, defined as the co-occurrence of two or more chronic conditions, is on the rise [5, 6]. Historically, low- and middle-income countries (LMICs) grappled more with infectious diseases, while NCDs and subsequent multimorbidity were considered to be the problems of high-income countries. However, recent evidence suggests that LMIC are also experiencing a high burden of multimorbidity. A scoping review on the prevalence of multimorbidity in LMICs reported prevalence ranging from 3.2% to 90.5% [7].

Literature on multimorbidity in the Nepali context is limited, and only two previous studies have reported a prevalence of approximately 14% [8, 9]. While these two are pioneer studies to shape our understanding of multimorbidity in Nepal, each has limitations. One of the studies was conducted in the rural areas of Nepal, while the other included a relatively younger population; only 14% of the participants were above 60 years of age. It is well established that age is strongly correlated with multimorbidity [10, 11], and in Nepal, NCDs risk factors are more concentrated in urban areas [12]. Hence, we anticipate that the findings from the two previous studies to be underestimated. Further, in previous studies from Nepal, minorities are represented in a small proportion. Thus, our proposed study supplements the previous studies to increase our understanding of multimorbidity among older adults, mostly from minority communities.

Additional study to enhance our understanding of multimorbidity among Nepali older adults is warranted from various perspectives. The health care system in Nepal is based on a single disease approach where each chronic condition is managed in siloes. Such a single disease approach may not be favorable to those with multiple conditions because on one side, the impact of multiple chronic diseases is greater than the cumulative effect of individual conditions, and on the other side, it requires a multitude of specialists’ referrals, biomedical investigations, and treatment [11]. People with multimorbidity have increased vulnerability to acute infections and pre-matured death [13], decreased quality of life [14], and are high utilizers of healthcare resources through more frequent hospital admissions, and longer hospital stays [15]. Especially in Nepal, where health resources are constrained, preventing the progression to the multimorbid stage is a financially viable option than treatment which usually involves complex, prolonged and expensive care [15]. Effective preventive strategies can be developed if the underlying epidemiology is well-understood. While multimorbidity moved onto the priority agenda for many health policymakers, it hasn’t been recognized as a significant health problem in Nepal. Notably, Nepal has recently acknowledged the burgeoning population of older adults and has expressed commitment to address their health, economic and financial needs [16]. Health care is an inevitable need in late life. Thus, to address the needs of burgeoning older adults with multimorbidity, the health care in Nepal should shift the paradigm from vertical mono-morbid approaches to horizontal multimorbid ones. Devising policies to address the health care needs requires understanding the epidemiology and identifying the at-risk groups for targeted intervention. Quantifying prevalence and identifying correlates is the first step to identify the burden and target groups. Hence, this study aims to assess the prevalence and correlates of multimorbidity among Nepali older adults in eastern Nepal.

## Methods

### Study design and participants

Data were obtained from a community-based cross-sectional survey of 847 Nepali older adults, aged 60 years and older, in three districts of Eastern Nepal between July and September 2020. The methodology is detailed elsewhere [17]. Briefly, the sample size of 847 was calculated based on unknown prevalence = 50%, CI = 95.0%, sampling error = 5.0%, design effect = 2, and non-response rate = 5.0%. Participants were recruited using multi-stage cluster sampling techniques from the Morang, Pachthar, and Terathum districts of Province 1 in Eastern Nepal. For inclusion in the study, participants were required to be Nepali nationals, 60 years or older, and have been residing in the study area for at least one year. Those that were excluded were residents of nursing facilities, with mental health conditions, with hearing impairment, or with an inability to communicate.

### Data collection and study variables

The data was collected via semi-structured interviews by twelve local healthcare providers employed by the Government of Nepal. Surveyors were oriented on various aspects of the study prior to data collection. All questionnaire materials were translated from English into Nepali and then verified through back-translation. The Nepali version of the tool was pilot tested among ten older adults who were excluded from the final data analysis. Interviews were conducted in the Nepali language at participants' households using Kobo Toolbox mobile app.

#### Dependent variable measurement

The presence of multimorbidity was the primary outcome of interest. Multimorbidity was measured on a binary scale, with it being either present or absent. The presence of multimorbidity was indicated by the presence of two or more morbidities [5]. Participants who had zero or one morbidity were indicated to have an absence of multimorbidity. The morbidities included in this study were hypertension, heart diseases, stroke, hypercholesterolemia, diabetes, chronic respiratory diseases, chronic kidney disease, cancer, and osteoarthritis. For each of these conditions, participants were asked if they had a given health condition diagnosed by a health professional and/or if they were taking any prescription medications for that condition. In multiple response type questions, participants selected all the applicable conditions. Finally, via an open-ended follow-up question, they were asked to specify any other conditions that were not captured. Each morbidity was classified as either present or absent and summed up to get a cumulative morbidity scale which was used to create dichotomized multimorbidity variable.

#### Independent variable measurement

The independent variables included in this analysis were age group, sex, marital status, ethnicity, education, urban/rural residence, current occupation, walking proximity to a health center, facing financial hardship with health care, recipient of social security allowance, and knowledge of senior citizen services. Health behaviors such as smoking, tobacco use, alcohol use, and physical activity were also included. Each of these variables, measured via self-report, is presented as categorical with responses as shown in Table 1. Details of these variables are provided in our previous study [17].

**Table 1:** Socio-Demographic and Health-related Characteristics of the Study Participants—Overall and by Multimorbidity Status

Multimorbidity				
	Overall (*n* = 843)	Present (*n* = 192)	Absent (*n* = 651)	*p*-value
	**n (%)**	**n (%)**	**n (%)**	
**Age in years**				
** 60–69**	383 (45.4)	44 (22.9)	339 (52.1)	** < 0.001**
** 70–79**	315 (37.4)	92 (47.9)	223 (34.3)	
** 80 + **	145 (17.2)	56 (29.2)	89 (13.6)	
**Sex**				
** Female**	412 (48.9)	109 (56.8)	303 (46.5)	**0.013**
** Male**	431 (51.1)	83 (43.2)	348 (53.5)	
** Marital status**				
** Married**	639 (75.8)	123 (64.1)	516 (79.3)	** < 0.001**
^** 1**^**Without partner**	204 (24.2)	69 (35.9)	135 (20.7)	
**Ethnicity**				
** Brahmins/Chhetri**	266 (31.6)	74 (38.5)	192 (29.5)	**0.018**
** Minorities and others**	577 (68.4)	118 (61.5)	459 (70.5)	
**Education**				
** No formal schooling**	577 (68.4)	125 (65.1)	452 (69.4)	0.257
** Formal schooling**	266 (31.6)	67 (34.9)	199 (30.6)	
**Residence**				
** Rural**	370 (43.9)	59 (30.7)	311 (47.8)	** < 0.001**
** Urban**	473 (56.1)	133 (69.3)	340 (52.2)	
**Current occupation**				
** Agriculture**	400 (47.5)	83 (43.2)	317 (48.7)	0.232
** Non-agriculture**	169 (20.0)	37 (19.3)	132 (20.3)	
** Retired/Housewife**	274 (32.5)	72 (37.5)	202 (31.0)	
**Walking proximity to the nearest health center**				
** Less than 30 min**	272 (32.3)	49 (25.5)	223 (34.3)	**0.017**
** 30–60 min**	372 (44.1)	85 (44.3)	287 (44.1)	
** More than 60 min**	199 (23.6)	58 (30.2)	141 (21.6)	
**Financial hardships with health care**				
** No**	379 (45.0)	90 (46.9)	289 (44.4)	0.544
** Yes**	464 (55.0)	102 (53.1)	362 (55.6)	
**Receiving social security allowance**				
** No**	409 (48.5)	61 (31.8)	348 (53.5)	** < 0.001**
** Yes**	434 (51.5)	131 (68.2)	303 (46.5)	
**Knowledge of Senior Citizen Services**				
** No**	98 (11.6)	29 (15.1)	69 (10.6)	0.087
** Yes**	745 (88.4)	163 (84.9)	582 (89.4)	
**Smoking**				
** Never**	579 (68.7)	129 (67.2)	450 (69.1)	0.611
** Current/former**	264 (31.3)	63 (32.8)	201 (30.9)	
**Tabaco use**				
** Never**	612 (72.6)	151 (78.6)	461 (70.8)	**0.033**
** Current/former**	231 (27.4)	41 (21.4)	190 (29.2)	
**Alcohol use**				
** Never**	581 (68.9)	141 (73.4)	440 (67.6)	0.124
** Current/former**	262 (31.1)	51 (26.6)	211 (32.4)	
**Physical activity**				
** Inactive**	512 (60.7)	113 (58.9)	399 (61.3)	0.544
** Active**	331 (39.3)	79 (41.1)	252 (38.7)	

^1^Includes widowed/separated/never married; All *p*-values from a Chi-square test

### Ethics

The original study was approved by the ethics committee of Nepal Health Research Council (Ref# 150/2020). This paper consists of secondary data analysis using a fully de-identified dataset. As such, the analysis received ethics approval with exempt status from Miami University’s Institutional Review Board (03653e).

### Statistical analyses

The participants’ demographic, social, and health characteristics are displayed using frequency (%) and group differences tested using the Chi-square test. Stepwise selection, starting with all the variables specified in Table 1, selected the final adjusted logistic regression model based on Akaike Information Criterion (AIC) . The model with the lowest AIC was selected and was tested for multicollinearity issues using Variance Inflation Factor (VIF) criteria. All VIF were < 2 suggesting no multicollinearity issues. Unadjusted and adjusted odds ratios and their corresponding 95% confidence intervals (CI) for the odds of multimorbidity are displayed in Table 3. SAS 9.4 was used to run all statistical tests and analyses.

## Results

### Study participants’ characteristics

Four participants refused to participate; response rate of 99.5%. Participants’ characteristics are presented in Table 1. A majority of the participants were in their sixties (45.4%) (Table 1). The participants were divided almost equally into male and female, with 48.9% women and 51.1% men. Individuals of minority and other ethnicities made up more than half of the participants in the study (68.4%), whereas the rest were composed of the Brahmin/Chhetri. Most participants in the study had no formal schooling (68.4% vs. 31.6% formal schooling), lived in an urban residence (56.1% vs. 43.9% rural residence), and were married (75.8% vs. 24.2% unmarried). The majority of participants also had financial hardship associated with healthcare (55% vs. 45%) and had knowledge of senior services (88.4% vs. 11.6%). The participants were almost equally divided into those receiving social security allowance (51.5%) and those who did not (48.5%). Most of the participants in the study lived within 30–60 min of walking distance to the nearest health center (44.1%) and never smoked (68.7%), used tobacco (72.6%) and alcohol (68.9%).

### Prevalence of individual chronic conditions/conditions and multimorbidity

Prevalence-wise, hypertension, osteoarthritis, chronic respiratory diseases, with an overall prevalence of 31.6%, 28.6%, and 18%, respectively, were the most common morbidities (Table 2). Participants had between 1–4 morbidities, with 66.5% of participants had at least single morbidity, and 22.8% had multimorbidity (Table 2).

**Table 2:** Prevalence of Individual Chronic Conditions and Multimorbidity

	**n (%)**
Single chronic conditions	
Hypertension	266 (31.6)
Osteoarthritis	241 (28.6)
Chronic Respiratory Disease	152 (18.0)
Diabetes	114 (13.5)
Heart Disease	45 (5.3)
Number of chronic diseases	
0	282 (33.4)
1	369 (43.8)
2	136 (16.1)
3	47 (5.6)
4	9 (1.1)
Multimorbidity status	
None (0–1 condition)	651 (77.2)
Multimorbidity (≥ 2 conditions)	192 (22.8)

A significant difference in the prevalence of multimorbidity was noted by various socioeconomic and lifestyle characteristics (Table 1). The prevalence of multimorbidity was significantly higher among older age groups and women compared to men (56.8% vs. 43.2%). The Chi-square test showed that the prevalence of multimorbidity was significantly different by participants’ marital status, ethnicity, residence, proximity to the health facility, recipient of social security allowance, and tobacco use (Table 1).

### Factors associated with multimorbidity

Based on the AIC criteria, the final adjusted model included age, sex, marital status, ethnicity, education, rural/urban residence, walking proximity to the nearest health center, receiving social security allowance, and knowledge of services for senior citizens (Table 3). Of these, only age, marital status, urban/rural residence, walking proximity, and knowledge of senior services were significantly associated with multimorbidity in the adjusted model. The odds of multimorbidity increased with age; those in their 70s had three times (adjusted odds ratio [AOR]: 3.11; 95% CI: 1.87–5.18) and those 80 plus had four times (AOR: 4.19; 95% CI: 2.32–7.57) increased odds of multimorbidity than those in their sixties. Individuals without a partner had 52% higher odds of multimorbidity than those married (AOR: 1.52; 95% CI: 1.00 to 2.30) with notable statistical significance at the borderline. Individuals who lived in urban areas had 71% higher odds of multimorbidity than individuals who lived in rural areas (AOR: 1.71; 95% CI: 1.16–2.51). Compared to participants who lived in proximity (less than 30 min walking), those living distant from the health facility (more than 60 min of walking) had 66% higher odds of multimorbidity (AOR: 1.66; 95% CI: 1.04–2.64). Participants who had knowledge of senior citizen services had 42% lower odds of multimorbidity than those who did not know about such services (AOR: 0.58; 95% CI: 0.34–0.97).

**Table 3:** Unadjusted and Adjusted Odds Ratios from Binary Logistic Regression for the Presence of Multimorbidity

	**Unadjusted Odds Ratio (95% CI)**	**Adjusted Odds Ratio (95% CI)**
**Age in years**		
** 60–69**	Ref	Ref
** 70–79**	**3.18 (2.14—4.73)**	**3.11 (1.87–5.18)**
** 80 + **	**4.85 (3.06—7.67)**	**4.19 (2.32–7.57)**
**Sex**		
** Female**	Ref	Ref
** Male**	**0.66 (0.48- 0.92)**	0.71 (0.47–1.07)
**Marital status**		
** Married**	Ref	Ref
^ **1**^**Without partner**	**2.14 (1.51—3.04)**	**1.52 (1.00–2.30)**
**Ethnicity**		
** Minorities and others**	Ref	Ref
** Brahmins/Chhetri**	**1.50 (1.07—2.10)**	0.95 (0.65—1.39)
**Education**		
** Formal schooling**	Ref	Ref
** No formal schooling**	0.82 (0.58—1.15)	0.69 (0.47–1.02)
**Residence**		
** Rural**	Ref	Ref
** Urban**	**2.06 (1.46—2.91)**	**1.71 (1.16–2.51)**
**Current occupation**		
** Retired/Housewife**	Ref	Not selected in the final model
** Agriculture**	0.73 (0.51—1.05)	
** Non-agriculture**	0.79 (0.50—1.24)	
**Walking proximity to the nearest health center**		
** Less than 30 min**	Ref	
** 30–60 min**	1.35 (0.91—2.00)	1.02 (0.67–1.55)
** More than 60 min**	**1.87 (1.21—2.89)**	**1.66 (1.04–2.64)**
**Financial hardships with health care**		
** No**	Ref	Not selected in the final model
** Yes**	0.90 (0.66—1.25)	
**Receiving social security allowance**		
** No**	Ref	Ref
** Yes**	**2.47 (1.75—3.47)**	1.07 (0.66–1.74)
**Knowledge of Senior Citizen Services**		
** No**	Ref	Ref
** Yes**	0.67 (0.42—1.06)	**0.58 (0.34–0.97)**
**Smoking**		
** Never**	Ref	Not selected in the final model
** Current/former**	1.09 (0.78—1.54)	
**Tabaco use**		
** Never**	Ref	Not selected in the final model
**Current/former**	**0.66 (0.45—0.97)**	
**Alcohol use**		
** Never**	Ref	Not selected in the final model
** Current/former**	0.75 (0.53—1.08)	
**Physical activity**		
** Inactive**	Ref	Not selected in the final model
** Active**	0.90 (0.65—1.25)	

^1^Includes widowed/separated/never married; statistically significant odds ratios are bolded

## Discussion

This study draws our attention to the prevalent multimorbidity among older adults in Eastern Nepal. Approximately one in five had multimorbidity, higher than the previously estimated prevalence of ~ 14% [9] from rural Nepal and among younger population [8, 9]. High prevalence of multimorbidity in our study was expected given that the risk factors for NCDs and subsequent burden of single NCDs are escalating in Nepal. Further, we anticipated the previous estimates to be underestimated, given the settings and populations of previous studies. In Nepal, NCDs risk factors are more concentrated in urban areas [12], and the strong correlation between age and multimorbidity is well-established [10, 11]. Hence, previous studies conducted among the younger population and rural residents were expected to report lower prevalence than ours. However, our estimate is lower than the 33% pooled prevalence reported by a meta-analysis of 70 community-based observational studies from 49 different countries [18]. Another review on the prevalence of multimorbidity among older adults in LMICs reported a wide variation in the estimated prevalence, ranging from 27.3% to 90.5% [7]. In addition to the differences in population characteristics, the literature suggests that methodologies employed to define and measure multimorbidity vary widely and contribute to varying prevalence estimates. Hence, there is a need for standardized definition and measurement of chronic multimorbidity and the list of conditions.

### Correlates of multimorbidity

Findings showed that the odds of multimorbidity increased with increasing age range, which is supported by the previous studies [7, 11, 19]. As individuals age, they have an increased probability of acquiring more morbidities due to cumulative exposures and accumulation of NCD’s risk factors across the life-course, in addition to biological senescence and declining physiology [20, 21]. Those who have lived longer have a greater chance of acquiring two or more such morbidities and consequently developing multimorbidity [22].

The study depicted that a higher percentage of females than males had multimorbidity [7]. This might be attributable to the longer chances of survival for females [22–24]; due to the accumulation of chronic conditions with age [6, 10, 11], those surviving longer are therefore likely to experience multimorbid conditions. Higher health care seeking and health service utilization by females [25] may result in higher diagnosis rates for females. In the patriarchal society of Nepal, in addition to gender biases since childhood, older women often face multiple disadvantages that may be particularly consequential for their health [26].

Married participants had lower odds of multimorbidity than those unmarried or without a partner. Our finding is supported by a longitudinal study aimed to assess the association between marital relationships and multimorbidity across multiple nations [27]. The health benefits of marriage could be attributed to the social and financial support received from the spouse [28]. Married older adults have better health outcomes because of their spouse's support, such as encouragement and reminders to take medicines, medical appointments, and companionship during health care visits. Optimum functioning and survival may be enhanced with the support of a partner in morbid conditions [29]. Additionally, they have higher socioeconomic status [30] that contributes to healthy aging. On the other hand, unmarried or participants without a spouse are more liberal in social life and may indulge in smoking and alcohol consumption [31–33]. Also, they may encounter stress because of daily life circumstances [34]. From the stress theory of health, these factors may contribute to adverse health, including the development of multimorbidity.

Participants living in urban areas had higher odds of multimorbidity than those living in rural areas, which is consistent with the previous multimorbidity study from Nepal [8]. Two factors explain the observed differences. First and most important is the fact that risk factors of NCDs such as smoking, alcohol, sedentary life, processed foods, etc., are more prevalent in urban Nepal [35]. A secondary explanation is that healthcare is more available and accessible in urban Nepal [36, 37]. Additionally, health care access and utilization are higher in urban Nepal. Thus, urban residents are more likely to be diagnosed with NCDs, given their higher health care utilization. Since this study relied on self-report, our participants from urban areas were more likely to be aware of their condition. To manage the high burden of multimorbidity in urban areas, it is necessary to design interventions promoting healthier lifestyle modification among the urban residents. Likewise, health care facilities in urban and rural areas should be better equipped, both in terms of human resources and physical infrastructure, which will help in early diagnosis and intervention.

The study showed lower odds of multimorbidity among those knowledgeable of senior citizen services. The Government of Nepal has been providing services tailored for senior citizens such as free essential healthcare services, subsidy on government-run health insurance, provision of old age allowance after the age of 70 years, discount on health treatment, and concession on transportation [25]. Older adults who are aware of free essential health services intend to utilize them more frequently and vice versa [25]. In addition, knowledge of services enables better health care decisions, early treatment, and compliance with treatment instructions, which results in improved health status and quality of life [38].

### Implications

A shift in the paradigm in Nepal's health care system must take place, prioritizing horizontal integration of services for older adults across multiple diseases domains rather than vertical integration between primary and secondary care within single disease domains [39, 40]. Understanding the underlying factors is an important step and can lead to effective prevention and management strategies [19]. Future research with longitudinal design may help us to better understand shared biological and environmental causes of multimorbidity among older adults. Services and programs for the older population should be specifically tailored to their age category since they are heterogeneous [41]. Programs to prevent multimorbidity should focus on improving lifestyles and dietary behaviors in urban areas while improving health facility access in rural areas [42]. Aside from transportation difficulties, a disparity in the doctor-patient ratio exists between the capital city and outside areas [43]. Strengthening public health facilities at the provincial and local levels could be a viable strategy to address health disparity between urban and rural settings. Health promotion programs should be tailored at the primary care level where community nurses, female community health volunteers, and mother groups can play a pivotal role in implementing program activities at the community level. Importantly, the findings on the high burden of multimorbidity call for designing an integrated model of care for people with multimorbidity that can address their multifaceted needs. However, one needs to be aware that without sufficient funding mechanism and capacity of the health workers, integration of services may lead to deterioration of health service delivery. The study findings also highlight the need for national level policies to address burgeoning multimorbidity challenges for health system in Nepal and effectiveness of its implementation in improving quality of care and people reported outcomes.

### Strengths and limitations

Strengths of the study include a large sample size with more than 50% minority population and a high response rate of 99%. Further, surveyors were local health care providers trained in study methodology. One of the major limitations is that disease information was collected by self-report and may have ascertainment bias. Health care visit  in Nepal is not routine, and some individuals may not even know about their disease status until it gets severe and requires medical attention. Hence, the prevalence of the individual chronic condition and subsequent multimorbidity may be underestimated. The survey population consisted only of residents of three districts of Province 1; therefore, the generalizability of the findings to other Provinces can be questioned. As the study was cross-sectional in design, a causal relationship between multimorbidity and the covariates cannot be established.

## Conclusion

The study found that one in five participants had multimorbidity. Multimorbidity was associated with various factors such as age, marital status, rural/urban residence, and distance from nearest health facility.

## Data Availability

The datasets used and/or analyzed during the current study are available from the corresponding author on reasonable request.

## Abbreviations

NCD: Non-Communicable Disease
LMICs: Low – and middle – income countries
AIC: Akaike information criterion
VIF: Variance inflation factor
CI: Confidence Interval
